# The Green Microalga *Coelastrella thermophila* var. *globulina* (Scenedesmaceae, Chlorophyta) Isolated from an Algerian Hot Spring as a Potential Source of Fatty Acids

**DOI:** 10.3390/life12040560

**Published:** 2022-04-08

**Authors:** Soumia Boutarfa, Mohammed Mourad Senoussi, Daniel Gonzalez-Silvera, José Ángel López-Jiménez, Marina Aboal

**Affiliations:** 1 Laboratory of Biomolecules and Plant Breeding, Department of Nature and Life Sciences, Faculty of Exact Sciences and Nature and Life Sciences, University of Larbi Ben M’hidi, Oum El Bouaghi 04000, Algeria; senoussi.mohamed.mourad@univ-oeb.dz; 2 Laboratory of Algology, Department of Plant Biology, Faculty of Biology, Espinardo Campus, E-30100 Murcia, Spain; maboal@um.es; 3 Department of Physiology, University of Murcia, E-30100 Murcia, Spain; danigs@um.es (D.G.-S.); joseang@um.es (J.Á.L.-J.)

**Keywords:** biotechnology, *Coelastrella thermophila* var. *globulina*, fatty acids, green algae, hot springs

## Abstract

Screening microalgae from extreme environments, including hot springs, is an important research topic that has lately emerged. A thermophilic green alga was isolated from a north-eastern Algerian hot spring at a temperature of 63 °C, and its fatty acid (FA) profile was explored. The strain was cultivated in BBM medium at 35 °C in a 16:8 h light/dark cycle and 75 μM photons m^−2^ s^−1^. The morphological studies combined with phylogenetic analysis revealed that the isolate was *Coelastrella thermophila* var. *globulina* Q. Wang, H. Song, X. Liu, G. Liu and Z. Hu. The monounsaturated fatty acid (MUFA) content was 51.12%. The saturated fatty acid (SFA) and polyunsaturated fatty acid (PUFA) content, respectively, accounted for 27.01% and 21.87%. The main FA was oleic acid (18:1n–9), whose value was 35.95%, followed in decreasing order by palmitic acid (16:0) with 21.45%, linoleic acid (18:2n–6) with 14.38% and α-linolenic acid (18:3n–3) with 04.22%. The FA profile exhibited high total n–6 and n–3 PUFA values (15.80% and 5.76%, respectively). *Coelastrella thermophila* var. *globulina* is particularly interesting for producing n-6 and n-3 PUFA and is likely suitable for other biotechnological purposes. This is the first time that this taxon has been reported in hot springs. Other species can be expected to be reported, which emphasises the importance of the biodiversity of extreme habitats.

## 1. Introduction

Microalgae are a highly diversified group of photosynthetic microorganisms adapted to a wide range of ecological habitats. They can utilise solar energy to combine water and carbon dioxide to generate biomass, which can be used for numerous renewable purposes, such as the production of biodiesel or biochemical products of pharmaceutical interest [1,2,3,4].

Microalgae, including Chlorophyceae, inhabit environments ranging from freshwater to extreme habitats such as snow, desert sand and hot springs [5,6,7]. They have recently been paid increasingly more attention in biotechnology domains for producing lipid-based biodiesel and polyunsaturated fatty acids (FAs) thanks to their potential therapeutic and nutritional uses [8,9,10,11]. In addition to fats, a variety of valuable molecules, such as polysaccharides, pigments, antioxidants, antimicrobial, high-value bioactive substances and other chemical compounds, may also be extracted from them to be used for lots of commercial applications, including the cosmetics and pharmaceutics industries [3,8,11,12,13,14,15,16,17,18,19,20,21,22].

The genus *Coelastrella* (Chlorophyta, Scenedesmaceae) was reported by Chodat [23]. This genus is described as being unicellular or making few celled aggregates and is remarkable for its sculptured cell wall with several longitudinal ribs [19,24,25,26]. It has been reported from mainly subaerial and terrestrial habitats, and only rarely from freshwater [25,27]. Several species of this genus show a high propensity to accumulate carotenoids and FAs, especially n-6 and n-3 PUFA, and display good resistance to diverse stresses [19,20,21,22,25,26]. Some species have also been proposed for applications in bioremediation, nutrient removal and nanoparticle production [20,28,29].

Nowadays, increasing interest is being shown in hot springs because some authors have proposed that screening microbial diversity from these habitats may be crucial for comprehending their role in biogeochemical cycles and to determine their biotechnological potential [7,30,31,32]. The thermophilic microalgae that inhabit hot springs are particularly interesting for biologists as the genetic origin of thermophily is not fully understood [33]. Likewise, isolating indigenous microalgae strains from these extreme habitats is essential, and several of these species might be more valuable than commercially available strains [26,34].

Algeria has more than 240 hot springs, mostly across its north-eastern area, with variable physical and chemical parameters [35]. The present work was conducted as part of a study to screen thermophilic microalgae from Algerian hot springs, where the high diversity of microalgae is still unexplored. Thus, we isolated a green alga strain and determined its FA profile by focusing on future biotechnological applications.

## 2. Materials and Methods

### 2.1. Sampling Site and Physico-Chemical Analysis

The Meskhoutine-Guelma spring, located in north-eastern Algeria, was chosen for this study. The study area is semi-arid with annual precipitation of 546.04 mm. The yearly average temperature is 17.75 °C, with mean monthly temperatures ranging from 9.72 °C to 26.76 °C [36]. Algal samples were collected in a small sterile plastic container labelled with sample collection details, including the date, site name, location code and replicate number. The samples taken for the analysis were stored in an icebox (4 °C) in the dark. Measurements of water parameters, including pH, water temperature, conductivity and dissolved oxygen, were taken in the field with a portable multiparameter probe (HORIBA W-23 XD). The water samples for the hydro-chemical analysis were collected using 250 mL polyethylene bottles. The following mineral contents were measured: Mg^2+^, K^+^, Na^+^, Ca^2+^, Al^−^, Li^+^, NO_3_^−^, SO_4_^2−^, Cl^−^, F^−^ and Br^−^. Cation species were determined with an Atomic Absorption Spectroscopy (AAnalyst 600, Shelton, CT, USA). Anion groups were analysed in an Ion Chromatography autosampler (Dionex As40, Sunnyvale, CA, USA) [37,38].

### 2.2. Isolation and Enrichment Cultures

The cultivation and isolation of strains were carried out under sterile conditions in solid BBM (Bold’s basal medium) enriched with soil extract to ensure the growth and multiplication of the green microalga at 35 °C in a 16:8 h light/dark cycle and 75 μM photons m^−2^ s^−1^. After approximately 3 weeks, algal cells were repeatedly streaked and incubated in identical settings as those mentioned above until the pure isolate was obtained. Monoclonal cultures were selected to produce enough biomass for lipid extraction purposes. The strain was incubated inside 250 mL culture flasks containing the same medium with aeration using an aquarium pump which pumped 1.5 L air per min through a drip set under the same aforementioned conditions for periods lasting up to 3 weeks. The biomass was harvested by centrifugation [39] and frozen at −80 °C until analysed. The strain was deposited in the MAESE culture collection at the University of Murcia (Spain) as MAESE 20.61.

### 2.3. Morphological Identification

The morphological identification of the natural and cultivated materials (designated as S3A) was performed under a light Olympus (BX50) microscope (400–1000) and photographed with an Olympus (U-CMAD-2) camera. Algal cells’ size, chloroplast shape, wall thickness and surface ornamentation, reproduction mode, as well as the presence of pyrenoids and starch granules, were determined at both the 40× and 100× magnifications. Morphotypes were identified down to the species level based on the works conducted by Chodat [23], John [24], Wang et al. [25] and Goecke et al. [26].

### 2.4. Molecular Identification and Phylogenetic Analysis

Green alga S3A DNA was extracted from cell pellets’ culture material by the Cetyl-Trimethyl Ammonium Bromide (CTAB) extraction method [40]. PCR amplification was conducted in a 12.5 μL volume, which contained approximately 0.5 μL of template DNA, 2 µM of each primer, 0.2 mM of dNTP, two units of Taq DNA polymerase, the manufacturer’s buffer and ddH_2_O to the final volume, amplified using primer pairs SSU1004/ITS1DR; SSU301/SSU1147 and SSU1+SSU568 [41]. PCR reactions were performed in the following settings: 4 min at 94 °C, 35 cycles of 60 s at 94 °C, 60 s at 50 °C, 90–120 s at 72 °C, and a final 10 min extension step at 72 °C [39]. The PCR reaction was run on a thermocycler (Eppendorf Mastercycler Gradient, Hamburg, Germany). The negative control PCR was also conducted using the same primers, but without a DNA sample.

The resulting DNA sequences were separated by electrophoresis on 1% agarose gel and examined under ultraviolet light. Following the manufacturer’s protocol, the resulting products were purified and excised from agarose gel before being cleaned with a PCR purification kit: GenElute PCR clean-up (Sigma-Aldrich, St. Louis, MO, USA). Finally, the purified amplification products were sequenced at the Genomic Service of the University of Murcia with the BigDye terminator cycle sequencing reaction (Applied Biosystems, Foster City, CA, USA) using the same PCR primers as those mentioned earlier.

The sequences from our samples were checked against the sequences in the GenBank database via a BlastN search online and were then aligned with the related sequences using MUSCLE. The phylogenetic tree was constructed with MEGA (11.0.10) [42] by neighbour-joining (NJ) based on evolutionary distances [43]. The sequences generated by this research were submitted to the GenBank nucleotide database from the National Center for Biotechnology Information (NCBI) with accession number OM831388.

### 2.5. Lipid Extraction and Fatty Acids Quantification

Lipids were extracted following the technique proposed by Folch et al. [44]. The conversion of lipids into FA methyl esters (FAMEs) was performed by acid-catalysed transesterification of total lipids according to the method of Christie [45]. The total lipid samples were transmethylated overnight in 2 mL of 2% sulphuric acid in methanol (plus 1 mL of toluene to dissolve neutral lipids) at 50 °C. Methyl esters were extracted twice in 5 mL of hexane–diethyl ether (1:1, *v/v*) after neutralisation with 2 mL of 2% KHCO_3_, dried under nitrogen and redissolved in 1 mL of iso-hexane. FAMEs were separated and quantified by gas-liquid chromatography in an SP™ 2560 flexible fused silica capillary column (length 100 m, internal diameter 0.25 mm, film thickness 0.20 mm SUPELCO) in a Hewlett–Packard 5890 gas chromatograph. The 140 °C oven temperature was initially increased at a rate of 3 °C min^−1^ to 230 °C, followed by 2 °C min^−1^, and then to 240 °C to be held for 12 min. The injector and flame ionisation detector were set at 260 °C. Helium was used as the carrier gas at a pressure of 300 kPa. Peaks were identified by comparing their retention times to appropriate FAME standards from the Sigma Chemical Company (St. Louis, MO, USA). Each component’s data were reported as a percentage of total content [8,46].

## 3. Results

### 3.1. Physico-Chemical Characteristics of the Algerian Hot Spring

The temperature of the Meskhoutine spring water was 63 °C, with a neutral pH (7.08) and moderate electrical conductivity (0.23 mS/m) (Table 1). The main anions were Cl^−^ (318 mg/L) and HCO_3_^−^ (366 mg/L), while the main cations were Na^+^ (219 mg/L) and Ca^+^ (220 mg/L) (Table 1).

### 3.2. Species Description

**Figure 1:**
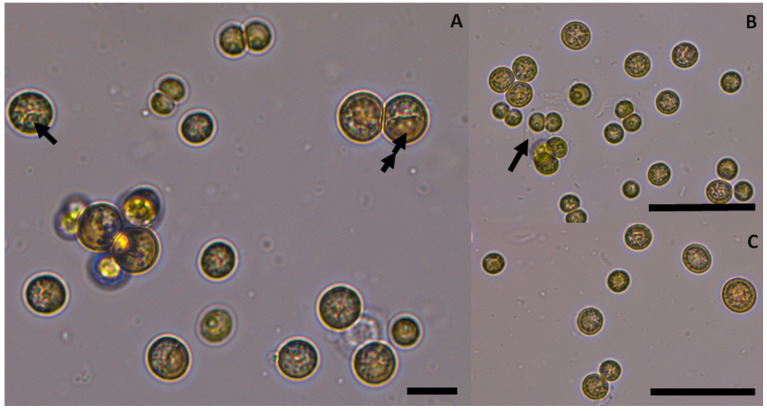
*Coelastrella thermophila* var. *globulina* in cultures: (**A**). Cells in various growth stages: a single arrow denotes pyrenoid and a double arrow indicates a big lipid droplet. (**B**). Mature cells and autospores: an arrow depicts cell wall remnants. (**C**). Autospores and mature cells. The scale in A represents 10 µm, with 50 µm in (**B**,**C**).

Based on the morphological features, isolate S3A was preliminarily identified as *Coelastrella* sp., characterised as unicellular and occasionally forming aggregates with vegetative cells spherical to subspherical, from 5.4–9 um in diameter, including a sole parietal and cup-shaped chloroplast that often rapidly changes shape and fragments into blades as cells age. Before cell division, mature cells are almost spherical and measure (8.5) 9.7–14 (18.5) um in diameter with conspicuous thickenings on cell walls. A distinct pyrenoid was clearly observed in vegetative cells and autospores. The longitudinal ribs on the cell wall of living cells are almost invisible in light microscopy. Reproduction was performed exclusively by autospores (4–16 elongated autospores in the sporangium cell). Aged cells frequently change colour to become brick-red/orange (Figure 1).

### 3.3. Phylogenetic Identification

**Figure 2:**
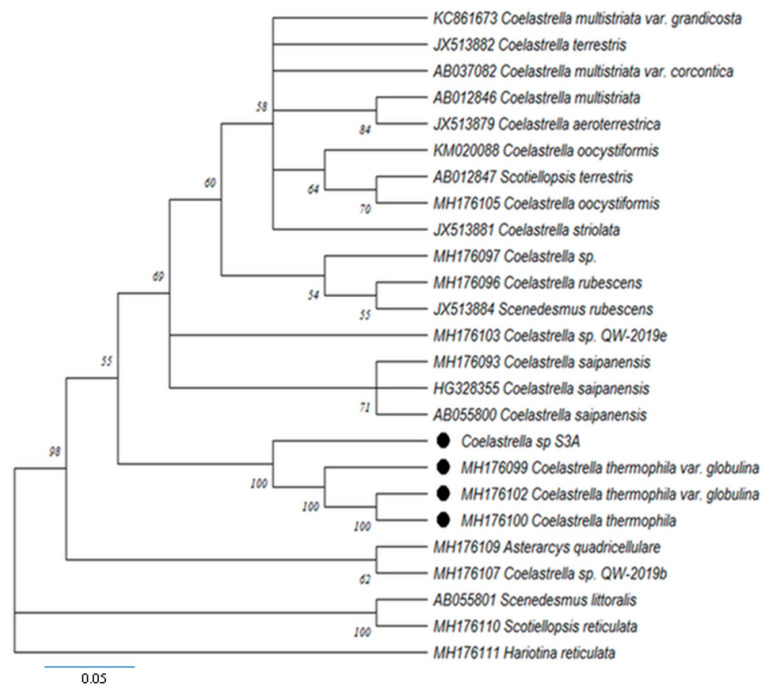
Phylogenetic tree of the studied *Coelastrella* sp. S3A strain reconstructed using a dataset of 25 18S rDNAs deriving from the genus *Coelastrella* and its relative species in Chlorophyceae. The bootstrap support percentages are shown near the corresponding nodes; the names of species and the strain, as well as the GenBank accession numbers, are shown.

A phylogenetic analysis based on the 18S rRNA gene sequence and a comparison to similar strains in the GenBank database indicated that the strain had a high similarity with other strain sequences of *Coelastrella* (Scenedesmaceae, Chlorophyceae) (Figure 2). The 18S rDNA phylogeny included 24 taxa. The phylogenetic tree showed a well-supported clade of *Coelastrella* which contained the genera *Coelastrella* and Asterarcys (Figure 2). *Coelastrella* S3A formed a sister group with *Coelastrella thermophila* var. *globulina*, as recently described by Wang et al. [25], and the branch have high support values (Figure 2).

### 3.4. Fatty Acid Composition

The FA contents of the *Coelastrella* *thermophila* var. *globulina* strain are found in Table 2 compared to other published *Coelastrella* strains. The FA profile in our isolate consisted of a high proportion of monounsaturated fatty acids (MUFA) (51.12% ± 0.12), 27.01% ± 0.09 of saturated fatty acids (SFA) and 21.87% ± 0.05 of polyunsaturated fatty acids (PUFA) (Table 2). Overall palmitic acid (16:0) was the predominant SFA (21.45% ± 0.08), oleic acid (18:1n−9) was the most prevalent MUFA (35.95% ± 0.07) and linoleic acid (18:2n−6) was the most abundant PUFA (14.38% ± 0.03). Another PUFA of interest to show a relatively high proportion of the total was α-linolenic acid (18:3n−3) (04.22% ± 0.07), while 20:4n−6 (araquidonic acid, ARA) and 18:3n−6 (γ-linolenic acid, GLA) had only 0.13% and 0.96%, respectively. Eicosapentanoic acid (20:5n−3, EPA) and docosahexaenoic acid (DHA) were not detected (Table 2).

## 4. Discussion

The temperature of the Meskhoutine-Guelma spring water is high (63 °C) with a chemical composition that is characteristic of the so-called mineral springs [48]. This spring has been used for therapeutic and bathing purposes because its water is not suitable as drinking water or for agriculture activities [35,49]. The eukaryotic thermophilic organisms growing in these hot springs have been poorly studied but may provide new and interesting chemical compounds to be biotechnologically obtained, and to enhance the value of these habitats in conservation [50].

The green microalga isolated from submerged stones morphologically fitted *Coelastrella thermophila* var. *globulina*, which has been recently described by Wang et al. [25]; 18S rDNA sequencing and the BLAST analysis confirmed its identification. *Coelastrella* S3A was clustered together with *Coelastrella thermophila* var. *globulina*, with good support, and this clade was consistent with the morphological data. There was a 100% similarity between the *Coelastrella* S3A sequence and the sequences of the material of *Coelastrella thermophila* var. *gobulina* uploaded to GeneBank by Wang et al. [25]. However, this genus has a complicated taxonomic history, which has been revised a number of times on the basis of morphological or molecular data [25].

The taxon has been previously reported from wet concrete floors or stones submerged in rivers. Algal cells are initially dark green but become brick-red or orange as they age, which indicates increased carotenoids synthesis [20,26,51]. Changes in chloroplast shape as cells age have also been reported in other *Coelastrella* strains [19,25]. To the best of our knowledge, neither *Coelastrella* nor any species or variety has been reported previously from hot springs in Algeria or elsewhere.

The ability of algae to accumulate large amounts of FAs compared to other organisms is one of the qualities that allow them to live in extreme environments [48]. The isolated strain had a remarkable FA profile and could be a promising candidate for biotechnological purposes [20,21] because it presents a high proportion of oleic acid and a relatively high percentage of PUFA (Table 2). Comparable FA percentages have been reported in thermotolerant strain *Coelastrella* sp. F50 with 26.25% of SFA and 41.47% of MUFA [16], and thermotolerant strain *Coelastrella* sp. FI69 with 27% of SFA, and a high value for MUFA [47]. They differed from other *Coelastrella* strains, such as freshwater *Coelastrella* sp. L3 with a high SFA value (62.97%) [10], and also *Coelastrella multistriata* [19] and terrestrial *Coelastrella* sp. FGS-001 [26], both of which contained a high proportion of PUFA (66.80% and 42.71%, respectively) (Table 2). However, *Coelastrella S3A* was able to generate valuable FA levels at a relatively high temperature (35 °C) under laboratory conditions compared to other *Coelastrella* strains, which were cultivated at relatively lower temperatures. All this makes this microalga an attractive option for FA production for biotechnological purposes [16,20]. The FA profile of the studied strain presented an average chain length that varied from C14 to C20, which is typical of microalgae (Table 2) [52]. It is usually contained in suitable feedstock for biodiesel production and guarantees high-quality biodiesel parameters [53,54]. The biodiesel generated from SFA and MUFA with a short-carbon chain are of higher quality. The studied strain exhibited relatively high proportions of SFA and MUFA, which qualifies it as a candidate for providing raw materials also for biodiesel production [48].

The main SFA was palmitic acid, the principal MUFA was oleic acid, while linoleic acid (LA) and α-linolenic acid (ALA) acids were dominant in PUFA (Table 2). Our findings are consistent with those reported for other *Coelastrella* strains (Table 2) [16,20,21,22,26,47]. The differences in FA composition of our strain under differing incubation conditions may promote the synthesis of one type or another and merit further studies.

Our *Coelastrella thermophila* var. *globulina* strain presented high contents of total n-6 and n-3 PUFA (Table 2), which is characteristic of *Coelastrella* strains [19]. The FAs from the n-6 and n-3 series are an essential complement in human and animal diets [8,52,55,56]. LA was also present and predominated in n-6 PUFA, while ALA was prominent in n-3 PUFA (Table 2). Therefore, the high linoleic and α-linolenic FA contents in our strain makes biomass a valuable nutritional supplement source and a prospective food additive for animals, where ALA and/or LA could meet essential FA requirements (Table 2) [22].

Although the percentages of GLA (18:3n-6, GLA) (0.96%) and stearidonic acid (18:4n.3, SA) (1.16%) were not high, the presence of these two FAs is interesting because they can metabolically promote ARA and EPA production in human cells, which are essential [52,57]. Likewise, the production of 0.96% GLA by the studied strain seemed relatively higher than the values recorded in other *Coelastrella* strains (Table 2) [10,16,19,26,47] and other thermophilic green microalgae [52]. *Coelastrella* S3A, isolated from an Algerian hot spring at a high temperature (63 °C), demonstrated the ability to grow in culture at a lower temperature (35 °C) than in its natural habitat. This thermal flexibility is important in investigative procedures because it enables not only research into the variability of FA content due to environmental factors, but also into the dynamic exploitation of this constituent [58].

## 5. Conclusions

The morphological features of the studied strain match those of the genus *Coelastrella* defined by Chodat in 1922, and the strain established a sister cluster with the newly defined *C. thermophila* var. *globulina* Q. Wang, H. Song, X. Liu, G. Liu and Z. Hu. The *Coelastrella* S3A strain contains essential FAs of high commercial value, such as n-3 and n-6, among others, and presents an interesting FA profile for algal biotechnology at higher temperatures, particularly as a dietary supplement. This green alga also exhibits a suitable FA profile with high SFA and MUFA values, and the main FAs found were C16–C20, which can be used effectively as raw material for biodiesel production. More biodiversity studies at hot springs in Algeria and elsewhere are highly recommended for providing data to select microalgal strains with an interesting chemical composition. This is the first report of a *Coelastrella* strain from hot springs worldwide.

## Figures and Tables

**Table 1:** Physico-chemical characterisation of the water from the Algerian hot spring.

Meskhoutine Hot Spring	Meskhoutine
Locality	Guelma
Coordinates	36°36′0″ N 7°24′0″ E
Temperature °C	63.00
pH	7.08
Conductivity mS/m	0.23
Dissolved oxygen mg/L	0.63
Ca^2+^ mg/L	220.00
Mg^2+^ mg/L	33.56
Na^+^ mg/L	219.00
K^+^ mg/L	25.00
HCO_3_^−^ mg/L	366.00
F^−^ mg/L	2.70
Cl^−^ mg/L	318.00
Br_2_ mg/L	2.17
SO_4_^2−^ mg/L	367.00
NO_3_^−^ mg/L	0.36
SiO_2_ mg/L	54.90
Li^+^ mg/L	1.16
Fe mg/L	0.13

**Table 2:** Fatty acid contents of *Coelastrella thermophila* var. *globulina* strain S3A and comparison to other published *Coelastrella* strains (% wt).

Fatty Acids	*Coelastrella* *thermophila* var. *globulina*	*Coelastrella* sp. L3	*Coelastrella* sp. F50	*Coelastrella multistriata MZ–Ch23*	*Coelastrella* sp. *FGS-001*	*Coelastrella* sp. FI69	*Coelastrella striolata* var. *multistriata*	*Coelastrella* *Rubescens* *V 195*	*Coelastrella* sp. *BGV*
	This study	[10]	[16]	[19]	[26]	[47]	[20]	[21]	[22]
14:0	0.40	2.46	-	0.25	-	0.35	-	-	2.60
15:0	1.39	0.60	-	-	0.15	-	-	-	-
16:0	21.45	35.98	22.23	18.61	17.70	19.88	17.90	20.05	20.30
18:0	2.88	14.54	3.55	1.49	0.24	3.85	1.30	0.80	-
20:0	0.44	1.26	0.47	-	-	0.87	-	-	-
22:0	0.27	3.80	-	0.27	-	0.87	-	-	-
24:0	0.19								
Total SFA	27.01	62.97	26.25	21.16	18.68	34.00	18.90	22.00	22.90
15:1n−5	0.12	-	-	-	-	-	-	-	-
16:1n−9	4.67	-	-	-	-	-	-	-	-
16:1n−7	0.71	2.64	3.34	1.63	11.18	2.70	1.00	1.00	-
18:1n−9	35.95	19.53	36.47	8.05	22.60	31.43	13.10	6.80	26.00
18:1n−7	3.04	-	1.53	2.63	-	-	2.20	-	-
20:1n−9	6.45	1.21	0.43	-	0.21	-	-	-	-
22:1n−9	0.08								
24:1n−9	0.10								
Total MUFA	51.12	24.52	41.47	12.04	35.43	37.00	15.30	11.60	26.00
18:2n−6	14.38	6.24	13.57	7.32	7.12	14.99	22.70	20.80	15.60
18:3n−6	0.96	0.32	1.07	-	0.89	-	-	-	-
20:3n−6	0.27	-	-	-	-	-	-	-	-
20:4n−6	0.13	-	-	-	-	-	-	-	-
Totaln−6 PUFA	15.80								
18:3n−3	4.22	3.82	8.63	38.12	32.40	6.92	28.30	30.40	35.50
18:4n−3	1.16	-	1.33	-	-	-	-	-	-
22:5n−3	0.29	-	-	-	-	-	-	-	-
Totaln−3 PUFA	5.76								
Total PUFA	21.87	10.38	30.87	66.80	42.71	27.00	51.00	61.20	51.10

## Data Availability

Relevant data is contained within the article.
